# Hypereosinophilia in Solid Tumors—Case Report and Clinical Review

**DOI:** 10.3389/fonc.2021.639395

**Published:** 2021-03-24

**Authors:** Ewa Zalewska, Łukasz Obołończyk, Krzysztof Sworczak

**Affiliations:** Department of Endocrinology and Internal Medicine, Medical University of Gdańsk, Gdańsk, Poland

**Keywords:** hypereosinophilia, solid tumor, paraneoplastic syndrome, renal cell cancer, prognosis

## Abstract

**Background:**

Renal cell cancer may cause various paraneoplastic syndromes; however, paraneoplastic hypereosinophilia occurs exceedingly rare. Thus far, only two cases of clear cell renal cell carcinoma (CCRCC) associated with hypereosinophilia have been reported. In this paper, we present a case of paraneoplastic hypereosinophilia associated with renal cell carcinoma and a review of the reported cases of hypereosinophilia in solid tumors.

**Methods:**

The review is based on an electronic literature search performed in the PubMed database in September 2020 with the following key terms: eosinophilia & neoplasm; eosinophilia & cancer; eosinophilia & paraneoplastic syndrome. Papers were included based on screening the titles and/or abstracts. We also included the case of our patient in the analysis.

**Case presentation:**

A 68-year-old Caucasian female patient with recurrent CCRCC was admitted to our Clinic for exacerbating dyspnea and chest and right upper abdominal pain, accompanied by confusion. Preliminary blood tests showed an increased white blood cell count of 40,770/μl, and an increased eosinophil count of 6,530/μl indicating eosinophilia. Several tests were carried out to rule out the noncancer causes of hypereosinophilia. The temporal appearance of eosinophilia and the recurrence of CCRCC without any other apparent potential causes led to the diagnosis of paraneoplastic hypereosinophilia. Despite treating with high doses of corticosteroids, only a transient decrement in eosinophil count was observed along with further deterioration of the patient’s condition. The patient succumbed to the disease 6 months following the tumor surgery and 2 months after the diagnosis of hypereosinophilia and tumor recurrence.

**Conclusion:**

Our observations are in agreement with the majority of reports showing that the occurrence of eosinophilia following tumor resection may indicate a poor prognosis, tumor recurrence, and rapid disease progression.

## Introduction

Eosinophilia, which is characterized by an increase in the count of circulating absolute eosinophils above the normal level of 500/μl, commonly occurs secondary to allergy, parasitic infections, collagen vascular disease, or drug hypersensitivity. Hypereosinophilia is marked by an elevated absolute eosinophil count (AEC) of more than 1,500/μl. Hematologic malignancies caused by somatic mutation have been reported with clonal expansion of eosinophils. Therefore, primary hypereosinophilia should be taken into consideration in the differential diagnosis of high AEC (1). However, in solid tumors, hypereosinophilia is a rare phenomenon and is mainly associated with carcinomas arising from the mucin-secreting epithelium (e.g. bronchus, gastrointestinal tract) (2).

Prolonged activation of eosinophils may cause migration into the skin, airway, gastrointestinal tract, cardiac, and nervous system, where they may cause end-organ damage principally through the induction of thrombosis and fibrosis. Therefore, all patients with hypereosinophilia must be evaluated for organ dysfunction attributable to eosinophilia (1).

It should be highlighted that peripheral blood eosinophilia counts are not strongly correlated and predictive of tissue eosinophilia. Moreover, tumor-associated tissue eosinophilia (TATE) is considered favorable in colorectal, breast, and prostate cancers, conversely, tumor-associated blood eosinophilia (TABE) generally occurs once the tumor has spread and its presence often leads to poor prognosis (3).

In this paper, we present a case of paraneoplastic blood eosinophilia associated with clear cell renal cell carcinoma (CCRCC) and a review of the reported cases of hypereosinophilia in solid tumors. The review is based on an electronic literature search performed in the PubMed database in September 2020 with the following key terms: eosinophilia & neoplasm; eosinophilia & cancer; eosinophilia & paraneoplastic syndrome. Papers were included based on screening the titles and/or abstracts. So far, only two cases of CCRCC associated with TABE have been reported (4, 5).

## Case Presentation

A 68-year-old Caucasian female was admitted to our Clinic on August 24, 2020, for exacerbating dyspnea and chest and right upper abdominal pain, accompanied by confusion for 3 weeks.

Five months earlier, in March 2020, she underwent radical right nephrectomy due to a CCRCC (tumor stage: pT3a, pNx, R0, M0). During the primary diagnosis, the blood analysis showed a white blood cell (WBC) count of 7,450/μl (reference range: 4,000–7,000/μl) with 1.1% (reference range: 1–6%) of eosinophilic granulocytes.

A computed tomography (CT) scan performed in July 2020, 3 months after the nephrectomy, revealed a retroperitoneal tumor mass in the surgical bed with hepatic and diaphragmatic invasion, and right pleural metastases. Histopathological assessment of liver infiltrates confirmed the diagnosis of local CCRCC relapse. During the diagnosis of tumor recurrence, the blood analysis showed a WBC count of 17,560/μl with 12.8% of eosinophilic granulocytes (AEC: 2,250/μl).

When abdominal pain and dyspnea occurred, palliative treatment with dexamethasone, pantoprazole, and buprenorphine were initiated. Moreover, targeted therapy for CCRCC was planned for the patient, but due to the rapid deterioration of health condition and a further increase of eosinophilic granulocyte count, the patient was admitted to our Clinic of Internal Medicine.

The past medical history also revealed that the patient had hypertension well controlled on bisoprolol only and underwent skin-sparing right mastectomy in 2005 for breast cancer (mammography and ultrasonography examination of the breasts, performed in August 2020, excluded the tumor recurrence). The patient had no history of alcohol abuse, asthma, or other allergic diseases.

On the day of admission to our Clinic, on examination, the patient appeared alert with mild functional cognitive disorder. Tachypnea and dullness over the right lung, up to seven intercostal spaces, were observed with the absence of breath sound. Abdomen tenderness during palpations and mild edema in distal parts of the lower limbs were also noticeable. Preliminary blood tests showed an increased C-reactive protein level of 240 mg/l, a WBC count of 40,770/μl, marked eosinophilia with an eosinophil count of 6,530/μl, mild anemia with a hemoglobin level of 11.6 g/dl, and a normal platelet count of 346,000/μl. A CT angiography of the chest revealed right-sided pleural effusion, pneumonia, and pulmonary microembolism.

Thoracentesis was performed for pleural effusion, and treatment with a broad-spectrum antibiotic (intravenous) and low-molecular-weight heparin (subcutaneous) were initiated. At that time, dexamethasone was discontinued due to the suspicion of infection disease and in purpose to carry out the differential diagnosis of hypereosinophilia. Despite the treatment, a further increase in leukocytes up to 56,000/μl with 33% of eosinophilic granulocytes (AEC: 18,500/μl) was observed.

Paraneoplastic eosinophilia is mainly associated with hematologic malignancies, although there is fairly extensive literature about eosinophilia in solid tumors, which mainly include case reports (4–96) (**Table 2**).

Several tests were performed to rule out the noncancer causes of hypereosinophilia. There was no clinical or serologic evidence of an allergy, a parasitic infection, or vasculitis. Blood tests showed a normal total immunoglobulin E (IgE) level of 59.2 kUa/l (normal < 81 kUa/l), while specific IgE for *Aspergillus fumigatus* or antibodies against myeloperoxidase were not detectable. A bone marrow biopsy was performed which showed no evidence of leukemia. Fluorescent in situ hybridization showed no FIP1L1-PDGFRa fusion. The temporal appearance of eosinophilia and recurrence of CCRCC without any other apparent potential causes confirmed the paraneoplastic nature of hypereosinophilia in our patient.

**Figure 1:**
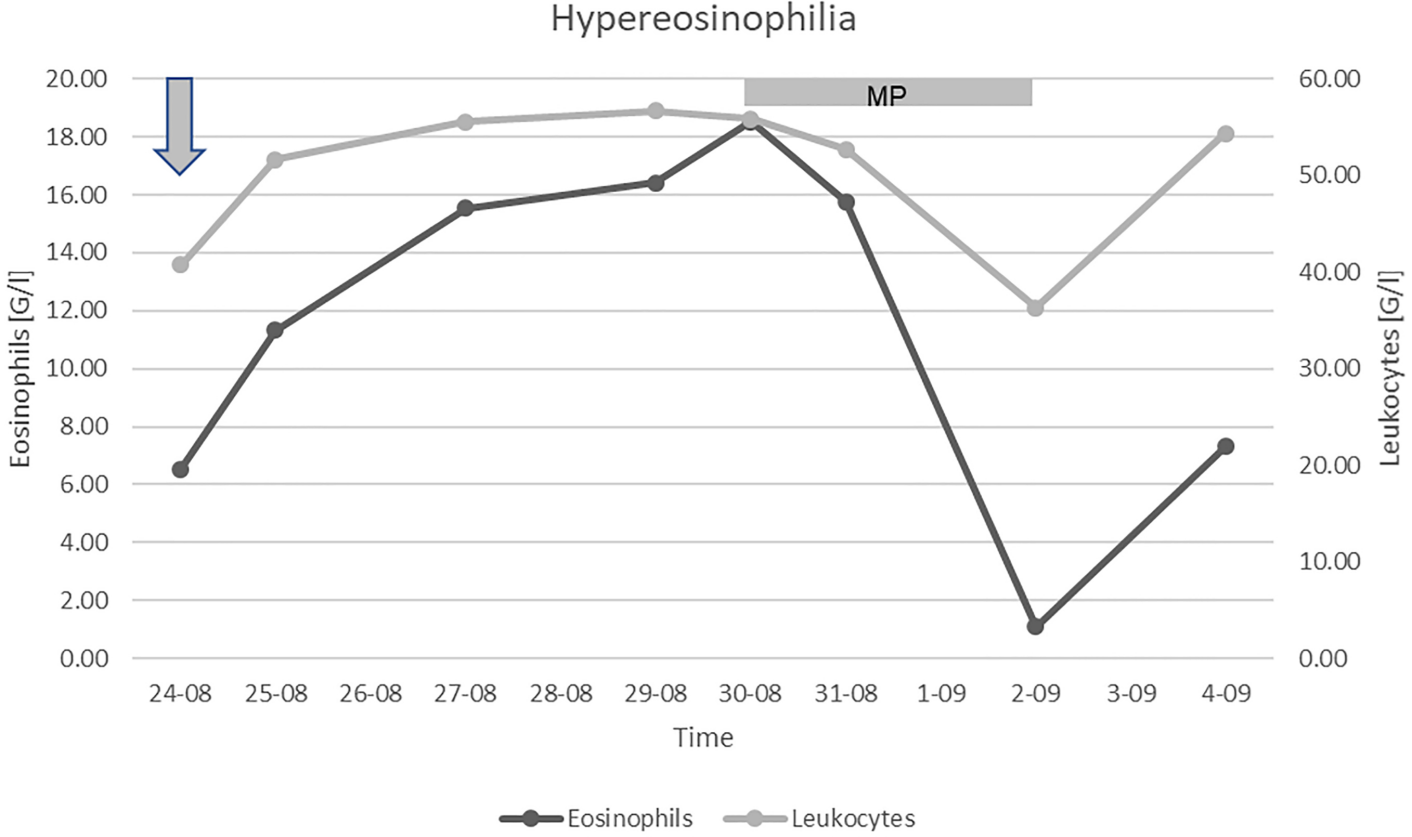
Leukocyte and eosinophilic granulocyte count. Arrow: admission to our Clinic, MP: treatment with methylprednisolone.

A massive intravenous dose of corticosteroids (1.5 g methylprednisolone) was initiated, which caused a profound, albeit transient, decrement in the eosinophil count (**Figure 1**). Unfortunately, the patient’s condition further deteriorated despite the treatment, and so targeted therapy for CCRCC was not initiated. We discontinued the corticosteroids after three days to limit their toxicity. Due to the poor prognosis, the patient received only palliative support and succumbed to the disease 6 months following surgery and 2 months after the diagnosis of tumor recurrence. Permission for a postmortem examination of the body was not granted.

## Discussion

In this paper, we have described the case of a patient with blood hypereosinophilia associated with recurrent CCRCC and analyzed one hundred previously reported cases of hypereosinophilia in solid tumors.

Renal cell cancer may cause various paraneoplastic syndromes, the most frequent of which are hypercalcemia, polycythemia, thrombocytosis, hypertension, and secondary amyloidosis (97). However, eosinophilia associated with renal cell cancer is exceedingly rare. So far, only two cases of CCRCC (4, 5) and three with chromophobe RCC associated with hypereosinophilia (27) have been reported (**Table 1**).

**Table 1:** Previously reported cases of renal cell carcinoma with hypereosinophilia.

	Age	Sex	AEC max [G/l]	Type	Tumor stage	Size [cm]	Recurrence	Follow-up
**Todenhöfer et al.** (4)	46	m	40	Clear cell RCC with sarcomatoid components	pT4, pNx, M1, L0, V1, Rx, G3	Not reported	Yes	Died of disease 4 months following surgery
**Wei et al.** (27)	53	m	17.5	Typical chromophobe RCC	T1b, N0, M0	7	No	1 year of follow-up
**Wei et al.** (27)	56	m	12.4	Typical chromophobe RCC	T1b, N0, M1	6.2	No	1 year of follow-up
**Wei et al.** (27)	48	f	19.1	Eosinophilic variant chromophobe RCC with sarcomatoid components	T2a, N0, M0	7.5	Yes	Died of disease 6 months following surgery
**Zhou et al.** (5)	75	m	78	Clear cell RCC	pT3a, pN1, M0, G4	2.5 × 1.7 × 1.3	Yes	Died of disease 2 months following surgery
**Our case**	67	f	18.5	Clear cell RCC	pT3a, pN1, M0, R0	10.8 × 10.3 × 11	Yes	Died of disease 8 months following surgery

AEC max—the highest absolute eosinophil count, RCC—renal cell carcinoma.

The mechanism contributing to eosinophilia in malignant diseases has not yet been fully determined. It has been suggested that three cytokines, namely interleukin-3 (IL-3), interleukin-5 (IL-5), and granulocyte-macrophage colony-stimulating factor (GM-CSF), may act as a potential eosinophilopoietin polypeptide (6, 13, 15, 24, 25, 29, 35, 50, 51, 76, 82, 83). Unfortunately, in our patient, neither the level of IL-5, IL-3, and GM-CSF in serum was determined nor immunohistochemical staining with indicated antibodies was performed.

Since eosinophilia occurs with several medical conditions, paraneoplastic eosinophilia can be diagnosed only after excluding all other causes. It is also necessary to rule out the following: infectious diseases (e.g. parasitic infections, allergic bronchopulmonary aspergillosis, coccidiomycosis); eosinophilias associated with medications (e.g. penicillins, cephalosporins, tetracyclines, sulfasalazine, nonsteroidal anti-inflammatory agents, hydrochlorothiazide, ranitidine, allopurinol, phenytoin, hydantoin, carbamazepine, cyclosporine, nevirapine); connective tissue and autoimmune diseases (e.g. eosinophilic fasciitis, eosinophilic granulomatosis with polyangiitis, sarcoidosis, bullous pemphigoid), and hematologic malignancies (e.g. chronic eosinophilic leukemia, B cell acute lymphoblastic leukemia, Hodgkin’s lymphomas) (1, 98).

For patients diagnosed with life-threatening conditions that may reflect irreversible eosinophil-associated tissue damage, emergency treatment with high doses of steroids, leukapheresis, and/or cytoreduction may be warranted (98). The mechanism by which corticosteroids induce a reduction in the eosinophil count remains obscure and proposed explanations, including cessation of bone marrow release of eosinophils; reduction of bone marrow eosinophil production and adhesion; eosinophil destruction; reversible sequestration of eosinophils in extravascular locations, and inhibition of eosinophil chemotaxis (99).

We have summarized the one hundred previously reported cases of paraneoplastic hypereosinophilia secondary to solid tumors in the years 1938 – 2020 in **Table 2** (4–96). We included in the table data of tumor location, sex ratio, average age, average maximal absolute eosinophil count, and average survival time after the diagnosis of high AEC.

**Table 2:** Data of one hundred previously reported cases of hypereosinophilia in solid tumors.

Tumor	No.	M:F	Age avg	AEC max [G/l]	AEC avg [G/l]	SVavg[Mo]	Reference
SCC of the lung	8	7:1	63	37.1	16.81	1.7	(6–11)
Adenocarcinoma of the lung	8	8:0	60	114.39	47.8	7.4	(6, 16, 38, 49, 60, 71, 82, 93)
Large cell carcinoma of the lung	7	6:1	64	125.58	62.36	2.8	(12–15, 17–19)
Not further defined NSCLC	5	5:0	64	139.5	52.52	8.2	(20–22)
Pleomorphic carcinoma of the lung	1	1:0	55	7.1	–	NR	(23)
Gastric adenocarcinoma	7	5:2	65	71.51	14.62	3	(61–67)
Adenocarcinoma of the colon	5	3:2	50	141.5	40.29	3.7	(68–70, 72, 73)
Adenocarcinoma of the rectum	2	1:1	71	6.1	5.43	NR	(92, 94)
Adenocarcinoma of the pancreas	3	2:1	66	44.12	17.14	2	(52–54)
PNETs	3	3:0	62	99.6	42.9	4.4	(56–58)
IPMN of the pancreas	1	0:1	72	3.74	–	2	(55)
ACC of the pancreas	1	1:0	59	1.8	–	2.5	(59)
CCRCC	3	2:1	63	78	45.5	2.7	(4, 5)*
Chromophobe RCC	3	2:1	52	19.08	16.32	NR	(27)
Spindle cell sarcoma of the kidney	1	1:0	76	7.77	–	NR	(77)
Anaplastic thyroid cancer	4	2:2	74	51.3	21.19	0.5	(44–47)
Undifferentiated thyroid carcinoma	2	0:2	78	37.39	20.14	0.9	(48, 50)
Papillary thyroid cancer	2	1:1	49	81.9	44.55	9.5	(51, 95)
Uterine leiomyosarcoma	4	0:4	59	175	45.72	NR	(37, 39–41)
Uterine leiomyomas	2	0:2	53	5.04	3.44	NR	(42)
Metastatic melanoma	4	3:1	57	105.79	76.77	1.3	(24–26, 28)
Hepatocellular carcinoma	3	2:1	60	21.43	12.51	1	(29–31)
Prostatic adenocarcinoma	3	2:1	74	55.44	21.12	NR	(78–80)
UCC of the bladder	2	1:1	55	8.38	6.24	NR	(33, 34)
UCC of the renal pelvis	1	0:1	83	26.24	–	NR	(32)
Gallbladder cancer	2	2:0	58	65.5	34.81	2.3	(35, 36)
SCC of the tongue	2	2:0	70	9.7	9.6	8	(90)
SCC of the maxillary sinus	1	1:0	46	2.1	–	NR	(91)
SCC of the cervix	1	0:1	42	4.74	–	3	(87)
Spindle cell sarcoma of the knee	1	0:1	41	77.79	–	NR	(76)
MFH of the knee	1	0:1	30	160.7	–	18	(85)
STS of the left elbow	1	0:1	67	38.18	–	0.3	(86)
Endometrioid ovarian carcinoma	1	0:1	88	15.38	–	NR	(43)
Cardiac rhabdomyosarcoma	2	2:0	40	14.2	11.41	NR	(74, 89)
Peritoneal mesothelioma	1	1:0	56	6.15	–	2	(88)
Adenocarcinoma CUP	2	0:2	65	9.3	8.1	1.5	(24, 96)
Anaplastic CUP	1	1:0	65	19.45	–	NR	(75)

No.—number of reported cases, M:F—male-to-female ratio, AEC max—the highest absolute eosinophil count, AEC avg—average absolute eosinophil count, SV [Mo] – average survival time after diagnosis of hypereosinophilia in months, NR – not reported or insufficient data to calculate the average survival time, SCC—squamous cell carcinoma, NSCLC—non-small-cell carcinoma, PNETs—pancreatic neuroendocrine tumors, IPMN—intraductal papillary mucinous neoplasm, ACC—acinar cell carcinoma, CCRCC—clear cell renal cell cancer, RCC—renal cell cancer, UCC—urothelial carcinoma, MFH—malignant fibrous histiocytoma, STS—soft tissue sarcoma, CUP—carcinoma of unknown primary, * - Our case

Among the one hundred previously reported and analyzed by us cases of TABE, the death of 68 patients was reported (4, 7, 8, 10–14, 16–22, 24–28, 30, 32, 34–37, 39, 44, 46–51, 53–60, 65, 67–69, 71, 72, 74–76, 82–88, 94–96). About 90% of the deaths occurred within one year after the diagnosis of paraneoplastic hypereosinophilia. Moreover, 9 of the remaining 32 patients were diagnosed with disseminated cancer and referred to palliative care (9, 38, 60, 62–64, 80, 89, 93). Resolution of peripheral eosinophilia following surgical resection was reported in 18 of the remaining 32 patients, although only ten patients were followed-up between 6 to 30 months (15, 23, 27, 33, 40–43, 61, 66, 73, 77, 78, 91, 92). The reappearance of eosinophilia may herald the onset of tumor recurrence. Therefore, serial estimations of WBC with differential count should be an integral part of the follow-up (17, 18, 22, 41, 85, 95).

In conclusion, our observations presented in this paper are in line with most studies reflecting that paraneoplastic blood hypereosinophilia is characterized by a more advanced disease and poor prognosis. In our opinion, in all patients with life-threatening eosinophil-associated organ damage, prompt treatment should be initiated (3).

## Data Availability Statement

The original contributions presented in the study are included in the article/supplementary material. Further inquiries can be directed to the corresponding author.

## Ethics Statement

The studies involving human participants were reviewed and approved by the Independent Bioethics Committee for Scientific Research at Medical University of Gdansk (NKBBN/622/2020). Written informed consent was not provided because the patient passed away. Written informed consent was obtained from her closest relative to publish the data.

## Author Contributions

EZ reviewed the literature, wrote the manuscript, and secured ethical approval for the study. ŁO and KS carried out critical interpretations. All authors contributed to the article and approved the submitted version.

## Conflict of Interest

The authors declare that the research was conducted in the absence of any commercial or financial relationships that could be construed as a potential conflict of interest.

## References

[1] Butt NM Lambert J Ali S Beer PA Cross NCP Duncombe A. Guideline for the investigation and management of eosinophilia. Br J Haematol (2017) 176(4):553–72. 10.1111/bjh.14488 28112388

[2] Beeson PB. Cancer and eosinophilia. N Engl J Med (1981) 309(13):792–3. 10.1056/NEJM198309293091310 6888457

[3] Sakkal S Miller S Apostolopoulos V Nurgali K. Eosinophils in Cancer: Favourable or Unfavourable? Curr Med Chem (2016) 23(7):650–66. 10.2174/0929867323666160119094313 26785997

[4] Todenhöfer T Wirths S Von Weyhern CH Heckl S Horger M Hennenlotter J. Severe paraneoplastic hypereosinophilia in metastatic renal cell carcinoma. BMC Urol (2012) 12(7):1–7. 10.1186/1471-2490-12-7 22436420 PMC3348004

[5] Zhou WW Guan YY Liu XM. Paraneoplastic eosinophilia in clear cell renal cell carcinoma. Chin Med J (Engl) (2015) 128(16):2271–2. 10.4103/0366-6999.162501 PMC4717976 26265628

[6] Sawyers CL Golde DW Quan S Nimer SD. Production of Granulocyte-Macrophage Colony-Stimulating Factor in Two Patients With Lung Cancer, Leukocytosis, and Eosinophilia. Cancer (1991) 69(6):1342–6. 10.1002/1097-0142(19920315)69:6<1342::AID-CNCR2820690607>3.0.CO;2-U 1540871

[7] Saliba WR Dharan M Bisharat N Elias M. Eosinophilic pancreatic infiltration as a manifestation of lung carcinoma. Am J Med Sci (2006) 331(5):274–6. 10.1097/00000441-200605000-00008 16702798

[8] Remacle P Bruart J Henneghien C. Bronchial cancer and hypereosinophilia. Eur Respir J (1988) 1(2):191–2. 3360093

[9] Majumdar NK Zahn DW. Pulmonary malignancy and eosinophilia; a discussion and case report. Am Rev Tuberc (1957) 75(4):644–7. 10.1164/artpd.1957.75.4.644 13411423

[10] Ramaiah RS Biagi RW. Eosinophilia: an unusual presentation of carcinoma of the lung. Practitioner (1982) 226(1372):1805–6. 7178018

[11] Grathwohl K LeBrun C Tenglin R. Eosinophilia of the blood. A search for the cause uncovers squamous cell carcinoma. Postgrad Med (1995) 97(3):169–170,172. 10.1080/00325481.1995.11945976 7877924

[12] Goffman TE Mulvihill JJ Carney DN Triche TJ Whang-Peng J. Fatal hypereosinophilia with chromosome 15q- in a patient with multiple primary and familial neoplasms. Cancer Genet Cytogenet (1983) 8(3):197–202. 10.1016/0165-4608(83)90135-8 6297705

[13] Watanabe M Ono K Ozeki Y Tanaka S Aida S Okuno Y. Production of Granulocyte-macrophage Colony-stimulating Factor in a Patient with Metastatic Chest Wall Large Cell Carcinoma. Jpn J Clin Oncol (1998) 28(9):559–62. 10.1093/jjco/28.9.559 9793030

[14] El-Osta H El-Haddad P Nabbout N. Lung carcinoma associated with excessive eosinophilia. J Clin Oncol (2008) 26(20):3456–7. 10.1200/JCO.2007.15.8899 18612162

[15] Pandit R Scholnik A Wulfekuhler L Dimitrov N. Non-Small-Cell Lung Cancer Associated With Excessive Eosinophilia and Secretion of Interleukin-5 as a Paraneoplastic Syndrome. Am J Hematol (2007) 82(3):234–7. 10.1002/ajh.20789 17160990

[16] Henry DW Rosenthal A McCarty DJ. Adenocarcinoma of the lung Associated with Eosinophilia and Hidebound Skin. J Rheumatol (1994) 21(5):972–3. 8064750

[17] Slungaard A Ascensao J Zanjani E Jacob HS. Pulmonary Carcinoma with Eosinophilia. N Engl J Med (1983) 309(13):778–81. 10.1056/NEJM198309293091307 6310397

[18] Kodama T Takada K Kameya T Shimosato Y Tsuchiya R Okabe T. Large Cell Carcinoma of the Lung Associated With Marked Eosinophilia. Cancer (1984) 54(10):2313–7. 10.1002/1097-0142(19841115)54:10<2313::AID-CNCR2820541044>3.0.CO;2-I 6091864

[19] Lammel V Stoeckle C Padberg B Zweifel R Kienle DL Reinhart WH. Hypereosinophilia driven by GM-CSF in large-cell carcinoma of the lung. Lung Cancer (2012) 76(3):493–5. 10.1016/j.lungcan.2012.02.014 22420949

[20] Verstraeten AS De Weerdt A Van Den Eynden G Van Marck E Snoeckx A Jorens PG. Excessive eosinophilia as paraneoplastic syndrome in a patient with non-small-cell lung carcinoma: A case report and review of the literature. Acta Clin Belg (2011) 66(4):293–7. 10.2143/ACB.66.4.2062571 21938985

[21] Knox AJ Johnson CE Page RL. Eosinophilia associated with thoracic malignancy. Br J Dis Chest (1986) 80:92–5. 10.1016/0007-0971(86)90017-3 3947528

[22] Barrett AJ Barrett A. Bronchial carcinoma with eosinophilia and cardiomegaly. Br J Dis Chest (1975) 69(0):287–92. 10.1016/0007-0971(75)90098-4 128374

[23] Fukutomi T Kohno M Izumi Y Watanabe M Hayashi Y Nomori H. Pulmonary pleomorphic carcinoma producing granulocyte-macrophage colony-stimulating factor: Report of a case. Surg Today (2012) 42(3):288–91. 10.1007/s00595-011-0043-2 22068679

[24] Stefanini M Motos RA Bendigo LL Claustro JC. Blood and bone marrow eosinophilia in malignant tumors. Role and nature of blood and tissue eosinophil colony-stimulating factor(s) in two patients. Cancer (1991) 68(3):543–8. 10.1002/1097-0142(19910801)68:3<543::AID-CNCR2820680317>3.0.CO;2-3 2065275

[25] Oakley SP Garsia RJ Coates AS. Eosinophilic leukaemoid reaction and interleukin-5 in metastatic melanoma. Med J Aust (1998) 169(9):501–1. 10.5694/j.1326-5377.1998.tb123384.x 9847906

[26] Rule SAJ Waterhouse P Costello C Retsas S. Paraneoplastic eosinophilia in malignant melanoma. J R Soc Med (1993) 86(5):295. 8505756 10.1177/014107689308600518 PMC1294010

[27] Wei YB Yan B Yin Z Yang JR. Chromophobe renal cell carcinoma associated with eosinophilia: A report of three cases. Exp Ther Med (2014) 8(1):91–4. 10.3892/etm.2014.1725 PMC4061223 24944603

[28] Siebenschein R Siebenmann RE. [Paraneoplastic eosinophilic leukemoid with eosinophilic parietal thromboendocarditis in malignant melanoma]. Schweiz Med Wochenschr (1977) 107(36):1257–65. 918585

[29] Balian A Bonte E Naveau S Foussat A Bouchet-Delbos L Berrebi D. Intratumoral production of interleukin-5 leading to paraneoplastic peripheral eosinophilia in hepatocellular carcinoma. J Hepatol (2001) 34(2):355–6. 10.1016/S0168-8278(00)00091-X 11281573

[30] Ranke EJ. Eosinophilia and Hepatocellular Carcinoma. New Ser (1965) 10(6):548–52. 10.1007/BF02233048 14295485

[31] Yuen BH Reyes CV Rawal PA Sosman J Jensen J. Severe eosinophilia and hepatocellular carcinoma: An unusual association. Diagn Cytopathol (1995) 13(2):151–4. 10.1002/dc.2840130215 8542796

[32] Scherer T. Tumor Associated Blood Eosinophilia and Eosinophilic Pleural Effusion: Case Report and Review of the Literature. Internet J Pulm Med (1999) 1(1):1–6.

[33] Uyar ME Turkay C Erbayrak M Koktener A. Eosinophilic colitis in a patient with advanced transitional cell carcinoma of the bladder: A paraneoplastic syndrome? Am J Med Sci (2008) 336(1):81–3. 10.1097/MAJ.0b013e31815adeda 18626244

[34] King R Fairbrother R Grant I Farrington K. Hypereosinophilia, Cardiomyopathy and Transitional Cell Carcinoma of the Bladder. Br J Urol (1992) 69(6):661–2. 10.1111/j.1464-410X.1992.tb15646.x 1638356

[35] Tsunematsu M Haruki K Uwagawa T Shiba H Yanaga K. Gallbladder cancer accompanied by uncontrollable eosinophilia: report of a case. Int Cancer Conf J (2020) 9(2):55–8. 10.1007/s13691-019-00395-1 PMC7109237 32257754

[36] Paryani J Gupta S Chaturvedi A Kumar V Akhtar N Suryavanshi P. Paraneoplastic Leukemoid Reaction in a Case of Carcinoma Gall Bladder: A Rare Scenario. J Carcinog Mutagen (2019) 10(1):1–2. 10.4172/2157-2518.1000332

[37] Bodon R Mijangos JA. Alkaline Phosphatase-Producing Leiomyosarcoma of the Uterus. Am J Surg (1972) 124(5):673–5. 10.1016/0002-9610(72)90111-0 5079806

[38] Motilal B Savita A Rajendra T. Peripheral eosinophilia in a case of adenocarcinoma lung: A rare association. J Assoc Chest Physicians (2020) 3(2):60–2. 10.4103/2320-8775.158859

[39] Ksienski D Cheung WY. Metastatic uterine leiomyosarcoma and eosinophilia. Obstet Gynecol (2011) 117(2):459–61. 10.1097/AOG.0b013e3181f683d2 21252788

[40] Onishi S Hojo N Sakai I Matsumoto T Watanabe A Miyazaki T. Secondary amyloidosis and eosinophilia in a patient with uterine leiomyosarcoma. Jpn J Clin Oncol (2005) 35(10):617–21. 10.1093/jjco/hyi156 16172171

[41] Pal L Parkash V Chambers JT. Eosinophilia and uterine leiomyosarcoma. Obstet Gynecol (2003) 101(5):1130–2. 10.1016/S0029-7844(02)02338-4 12738126

[42] Buka NJ. Eosinophilia Associated With Uterine Leiomyomas. Can Med Assoc J (1965) 93(4):163–5. PMC1928549 14323659

[43] Hasan Albitar HA Egan AM Alkhateeb H Almodallal Y Iyer VN. Marked hypereosinophilia secondary to endometrioid ovarian cancer presenting with asthma symptoms, a case report. Respir Med Case Rep (2020) 31:101178. 10.1016/j.rmcr.2020.101178 32775193 PMC7404536

[44] Camargos EF Pandolfi MB Toledo MAV Quintas JL Moreira S De Azevedo AEB. A 95-year-old woman with leucocytosis and eosinophilia: Anaplastic carcinoma in an ectopic thyroid. BMJ Case Rep (2010) 2823:1–5. 10.1136/bcr.03.2010.2823 PMC3028571 22767520

[45] Feffer J Aziz M Schulman R. Paraneoplastic hyperosinophilia and neutrophilia due to anaplastic thyroid carcinoma. Endocr Pract (2016) 22:294–5. 10.1016/S1530-891X(20)44832-3

[46] Van Crombrugge P Pauwels R Van der Straeten M. Thyroid carcinoma and eosinophilia. Ann Clin Res (1983) 15(3):128–30. 6638927

[47] Shiraishi J Koyama H Seki M Hatayama M Naka M Kurajoh M. Anaplastic thyroid carcinoma accompanied by uncontrollable eosinophilia. Intern Med (2015) 54(6):611–6. 10.2169/internalmedicine.54.3446 25786451

[48] Nakada T Sato H Inoue F Mizorogi F Nagayama K Tanaka T. The production of colony-stimulating factors by thyroid carcinoma is associated with marked neutrophilia and eosinophilia. Intern Med (1996) 35(10):815–20. 10.2169/internalmedicine.35.815 8933194

[49] Margolin ML Zeitlin N Friedman YE Globus O Mouallem M. Eosinophilia and leukocytosis in a patient with lung cancer. Isr Med Assoc J (2019) 21(1):58–9. 30685910

[50] Miller WM Adcook KJ Moniot AL Raymond LW Hutcheson J Elliott RC. Progressive hypereosinophilia with lung nodules due to thyroid carcinoma. Chest (1977) 71(6):789–91. 10.1378/chest.71.6.789 862454

[51] Vassilatou E Fisfis M Morphopoulos G Savva S Voucouti E Stefanoudaki K. Papillary thyroid carcinoma producing granulocyte-macrophage colony-stimulating factor is associated with neutrophilia and eosinophilia. Hormones (2006) 5(4):303–9. 10.14310/horm.2002.11196 17178706

[52] Ibrahim U Asti D Saqib A Mudduluru BM Ayaz S Odaimi M. Eosinophilia as the presenting sign in pancreatic cancer: an extremely rare occurrence. Postgrad Med (2017) 129(3):399–401. 10.1080/00325481.2017.1246345 27718779

[53] Miyagawa F Danno K Uehara M. Erythema gyratum repens responding to cetirizine hydrochloride. J Dermatol (2002) 29(11):731–4. 10.1111/j.1346-8138.2002.tb00211.x 12484436

[54] Hirata J Koga T Nishimural J. Pancreatic carcinoma associated with marked eosinophilia: A case report. Eur J Haematol (1987) 39(5):462–6. 10.1111/j.1600-0609.1987.tb01457.x 3500871

[55] Dregoesc MI Iancu AC Lazar AA Balanescu S. Hypereosinophilic syndrome with cardiac involvement in a patient with multiple malignancies. Med Ultrason (2018) 20(3):399. 10.11152/mu-1574 30167597

[56] Sagi L Amichai B Barzilai A Weitzen R Trau H. Pancreatic panniculitis and carcinoma of the pancreas. Clin Exp Dermatol (2009) 34(5):205–7. 10.1111/j.1365-2230.2008.02992.x 19077093

[57] O’boyle CP Otridge B Dempsey J Barniville H. Malignant islet-cell tumour of the pancreas presenting with non-bacterial thrombotic endocarditis and eosinophilia. Postgrad Med J (1981) 57(669):457–8. 10.1136/pgmj.57.669.457 PMC2424945 6273832

[58] Haldane JH Kapoor H Morris J. Severe eosinophilia associated with a malignant islet cell tumour. Can Med Assoc J (1989) 140(9):1061–3. PMC1268980 2539897

[59] Robertson JC Eeles GH. Syndrome Associated with Pancreatic Acinar Cell Carcinoma. Br Med J (1970) 2(5711):708–9. 10.1136/bmj.2.5711.708 PMC1700617 5429656

[60] Abughanimeh O Tahboub M Abu Ghanimeh M. Metastatic Lung Adenocarcinoma Presenting with Hypereosinophilia. Cureus (2018) 10(6):e2866. 10.7759/cureus.2866 30148018 PMC6107036

[61] Nomura T Kodama K Moriuchi R Yaosaka M Kawasaki H Abe M. Papuloerythroderma of Ofuji associated with early gastric cancer. Int J Dermatol (2008) 47(6):590–1. 10.1111/j.1365-4632.2008.03635.x 18477151

[62] Fridlender ZG Simon HU Shalit M. Metastatic carcinoma presenting with concomitant eosinophilia and thromboembolism. Am J Med Sci (2003) 326(2):98–101. 10.1097/00000441-200308000-00008 12920442

[63] Teoh SC Siow W Tan H. Severe Eosinophilia in Disseminated Gastric Carcinoma. Singapore Med J (2000) 41(5):232–4. 11063174

[64] Takagi A Ozawa H Oki M Yanagi H Nabeshima K Nakamura N. Helicobacter pylori-negative advanced gastric cancer with massive eosinophilia. Intern Med (2018) 57(12):1715–8. 10.2169/internalmedicine.0013-17 PMC6047998 29434119

[65] Takeda H Nishikawa H Tsumura T Sekikawa A Maruo T Okabe Y. Prominent hypereosinophilia with disseminated intravascular coagulation as an unusual presentation of advanced gastric cancer. Intern Med (2014) 53(6):563–9. 10.2169/internalmedicine.53.1483 24633025

[66] Funaki M Ohno T Dekio S Jidoi J Nakagawa C Kin S. Prurigo nodularis associated with advanced gastric cancer: Report of a case. J Dermatol (1996) 23(10):703–7. 10.1111/j.1346-8138.1996.tb02684.x 8973036

[67] Tsutsumi Y Ohshita T Yokoyama T. A case of gastric carcinoma with massive eosinophilia. Acta Pathol Jpn (1984) 34(1):117–22. 10.1111/j.1440-1827.1984.tb02189.x 6328861

[68] Kato H Kohata K Yamamoto J Ichikawa S Watanabe M Ishizawa K. Extreme eosinophilia caused by interleukin-5-producing disseminated colon cancer. Int J Hematol (2010) 91(2):328–30. 10.1007/s12185-010-0491-2 20131104

[69] Uemura K Nakajima M Yamauchi N Fukayama M Yoshida K. Sudden death of a patient with primary hypereosinophilia, colon tumours, and pulmonary emboli. J Clin Pathol (2004) 57(5):541–3. 10.1136/jcp.2003.015321 PMC1770288 15113865

[70] Anagnostopoulos GK Sakorafas GH Kostopoulos P Margantinis G Tsiakos S Terpos E. Disseminated colon cancer with severe peripheral blood eosinophilia and elevated serum Levels of interleukine-2, interleukine-3, interleukine-5, and GM-CSF. J Surg Oncol (2005) 89(4):273–5. 10.1002/jso.20173 15726608

[71] Nqwata L Wong ML Mohanlal RD Lakha AB. Hypereosinophilia as a paraneoplastic phenomenon in non-small cell lung carcinoma. South Afr Respir J (2015) 21(4):108–9. 10.7196/SARJ.2015.v21i4.38

[72] Brick IB Glazer L. Marked Eosinophilia with Cancer A Poor Prognostic Sign. Ann Intern Med (1951) 35(1):213–8. 10.7326/0003-4819-35-1-213 14847458

[73] Caruso AA Costigliola F Salzano J Del Prete S Marasco D Imperatore C. Nasal and systemic eosinophilia associated with solid intestinal tumors, a case report and review of the literature. Ann Ital Chir (2019) 8:1–5. 30837352

[74] Sullivan MJ Wanger GP Schonfeld SA Bashore TM. Cardiac rhabdomyosarcoma presenting as hypereosinophilic syndrome. Am J Cardiol (1983) 51(5):909–10. 10.1016/S0002-9149(83)80158-1 6829455

[75] Abali H Altundag MK Engin H Altundag ÖÖ Türker A Üner A. Hypereosinophilia and metastatic anaplastic carcinoma of unknown primary. Med Oncol (2001) 18(4):285–8. 10.1385/MO:18:4:285 11918455

[76] Snyder MC Lauter CB. Eosinophilic and neutrophilic leukemoid reaction in a woman with spindle cell sarcoma: a case report. J Med Case Rep (2010) 4(1):335. 10.1186/1752-1947-4-335 20964813 PMC2984466

[77] McNall A Drinker HJ. Spindle cell sarcoma of the kidney with associated eosinophilia. Q Bull Northwest Univ Med Sch (1959) 33(1):12–4. PMC3803691 13634328

[78] Ishiguro T Kimura H Araya T Minato H Katayama N Yasui M. Eosinophilic pneumonia and thoracic metastases as an initial manifestation of prostatic carcinoma. Intern Med (2008) 47(15):1419–23. 10.2169/internalmedicine.47.1124 18670149

[79] Paolini MV Jankilevich G Graziano C Fernández Romero DS. Allergic contact dermatitis to manganese in a prosthodontist with orthodontics. Allergol Immunopathol (Madr) (2010) 38(1):48–50. 10.1016/j.aller.2009.05.005 19836873

[80] Ashdhir P Jain P Pokharna R Nepalia S Sharma SS. Pancreatic Cancer Manifesting as Liver Metastases and Eosinophillic Leukemoid Reaction: A Case Report and Review of Literature. Am J Gastroenterol (2008) 103(4):1052–4. 10.1111/j.1572-0241.2007.01772_16.x 18397441

[81] Saito K Kuratomi Y Saito T Kuzuya T Yoshida S Moriyama S-I. Primary squamous cell carcinoma of the Thyroid associated with Marked Leukocytosis and Hypercalcemia. Eur Ann Otorhinolaryngol Head Neck Dis (1981) 48:2080–3. 10.1002/1097-0142(19811101)48:9<2080::AID-CNCR2820480927>3.0.CO;2-N 7296515

[82] Lo CH Jen YM Tsai WC Chung PY Kao WY. Rapidly evolving asymptomatic eosinophilia in a patient with lung adenocarcinoma causes cognitive disturbance and respiratory insufficiency: Case report. Oncol Lett (2013) 5(2):495–8. 10.3892/ol.2012.1020 PMC3573146 23420572

[83] Walter R Joller-Jemelka HI Salomon F. Metastatic squamous cell carcinoma with marked blood eosinophilia and elevated serum interleukin-5 levels. Exp Hematol (2002) 30(1):1–2. 10.1016/S0301-472X(01)00764-0 11823029

[84] Coşkun HŞ Er Ö Tanrıverdi F Altınbaş M. Hypereosinophilia as a Preclinical Sign of Tongue Squamous Cell Cancer in a Gastric Cancer Patient with Complete Remission. Turkish J Haematol (2003) 20(2):107–10. 27265441

[85] Ando J Sugimoto K Tamayose K Ando M Kojima Y Oshimi K. Cytokine-producing sarcoma mimics eosinophilic leukaemia. Eur J Haematol (2007) 78(2):169–70. 10.1111/j.1600-0609.2006.00787.x 17328718

[86] Latif N Zaiden R Pham D Rana F. Soft Tissue Sarcoma Mimicking Eosinophilic Leukemia. Clin Adv Hematol Oncol (2010) 8(12):899–901. 21326167

[87] Reddy SS Hyland RH Alison RE Sturgeon JF Hutcheon MA. Tumor-associated peripheral eosinophilia: two unusual cases. J Clin Oncol (1984) 2(10):1165–9. 10.1200/JCO.1984.2.10.1165 6387057

[88] Davis BH. Hypereosinophilia associated with a peritoneal mesothelioma. Arch Pathol Lab Med (1979) 103(9):487. 582381

[89] Lo Re V Fox KR Ferrari VA Scott CH Kossev PM Kostman JR. Hypereosinophilia associated with cardiac rhabdomyosarcoma. Am J Hematol (2003) 74(1):64–7. 10.1002/ajh.10373 12949893

[90] Weller PF Kilon AD. Approach to the patient with unexplained eosinophilia. In: Mahoney DH, Newburger P, Rosmarin AG, Feldweg AM, editors. UpToDate (2020). p. 1-8. Available from: https://www.uptodate.com/contents/approach-to-the-patient-with-unexplained-eosinophilia/print%0A.

[91] Sato M Yoshida H Yanagawa T Yura Y Sugi M Hamada S. Carcinoma of the maxillary sinus with eosinophilia: Report of a case. Int J Oral Surg (1981) 10(1):62–7. 10.1016/S0300-9785(81)80009-9 6792096

[92] Tajima K Yamakawa M Inaba Y Katagiri T Sasaki H. Cellular localization of interleukin-5 expression in rectal carcinoma with eosinophilia. Hum Pathol (1998) 29(9):1024–8. 10.1016/S0046-8177(98)90212-X 9744323

[93] Machaczka M Hubert J Kasina F Klimkowska M. Eosinophilia as a presenting symptom of the metastatic lung adenocarcinoma with an unknown primary localization. Cent Eur J Med (2011) 6(5):541–4. 10.2478/s11536-011-0048-7

[94] Inoue M Kadono J Sugita H Nakazono T Motoi S Kitazono I. Impact of chemotherapy on eosinophilia-associated advanced rectal cancer: A case report and review of the literature. Oncol Lett (2016) 12(6):5269–74. 10.3892/ol.2016.5364 PMC5228383 28105235

[95] Nagel LR. Eosinophilia in cancer. N Engl J Med (1956) 250(14):607. 10.1056/NEJM195404082501406 13154595

[96] Morgan WG Ballinger WM. An unusual cause for eosinophilic leukocytosis. J Am Med Assoc (1938) 110:952. 10.1001/jama.1938.62790130002005a

[97] Gray RE Harris GT. Renal cell carcinoma: Diagnosis and management. Am Fam Physician (2019) 99(3):179–84. 30702258

[98] Williams KW Ware JA Abiodun A Holland-Thomas NC Khoury P Klion AD. Hypereosinophilia in Children and Adults: A Retrospective Comparison. J Allergy Clin Immunol Pract (2016) 4(5):941–947.e1. 10.1016/j.jaip.2016.03.020 27130711 PMC5010485

[99] Altman LC Hill JS Hairfield WM Mullarkey MF. Effects of corticosteroids on eosinophil chemotaxis and adherence. J Clin Invest (1981) 67(1):28–36. 10.1172/JCI110024 7005265 PMC371568

